# High prevalence of reduced fertility and use of assisted reproductive technology in a German cohort of patients with peripartum cardiomyopathy

**DOI:** 10.1007/s00392-022-02034-x

**Published:** 2022-05-13

**Authors:** Tobias J. Pfeffer, Manuel List, Cordula Schippert, Bernd Auber, Melanie Ricke-Hoch, Valeska Abou-Moulig, Dominik Berliner, Johann Bauersachs, Denise Hilfiker-Kleiner

**Affiliations:** 1 Department of Cardiology and Angiology, Hannover Medical School, Hannover, Germany; 2 Division of Reproductive Medicine, Department of Obstetrics and Gynecology, Hannover Medical School, Hannover, Germany; 3 Department of Human Genetics, Hannover Medical School, Hannover, Germany; 4 Institute of Cardiovascular Complications in Pregnancy and in Oncologic Therapies, Medical Faculty of the Philipps University Marburg, Baldingerstraße, 35032 Marburg, Germany

**Keywords:** Peripartum cardiomyopathy (PPCM), ART, Subfertility, Risk factor, Heart failure

## Abstract

**Background:**

Over the past decades the use of assisted reproduction technology (ART) increased worldwide. ARTs are associated with an elevated risk for cardiovascular complications. However, a potential relation between subfertility/ARTs and the heart disease peripartum cardiomyopathy (PPCM) has not been systematically analyzed yet.

**Methods:**

A retrospective cohort study was carried out, including *n* = 111 PPCM patients from the German PPCM registry. Data from PPCM patients were compared to those from postpartum women in the German general population.

**Results:**

The prevalence of reported subfertility was high among PPCM patients (30%; 33/111). Most of the subfertile PPCM patients (55%; 18/33) obtained vitro fertilizations (IVF) or intracytoplasmic sperm injections (ICSI). PPCM patients were older (*p* < 0.0001), the percentage of born infants conceived by IVF/ICSI was higher (*p* < 0.0001) with a higher multiple birth (*p* < 0.0001), C-section (*p* < 0.0001) and preeclampsia rate (*p* < 0.0001), compared to postpartum women. The cardiac outcome was comparable between subfertile and fertile PPCM patients. Whole exome sequencing in a subset of *n* = 15 subfertile PPCM patients revealed that 33% (5/15) carried pathogenic or likely pathogenic gene variants associated with cardiomyopathies and/or cancer predisposition syndrome.

**Conclusions:**

Subfertility occurred frequently among PPCM patients and was associated with increased age, hormonal disorders, higher twin pregnancy rate and high prevalence of pathogenic gene variants suggesting a causal relationship between subfertility and PPCM. Although this study found no evidence that the ART treatment per se increases the risk for PPCM or the risk for an adverse outcome, women with subfertility should be closely monitored for signs of peripartum heart failure.

**Graphical abstract:**

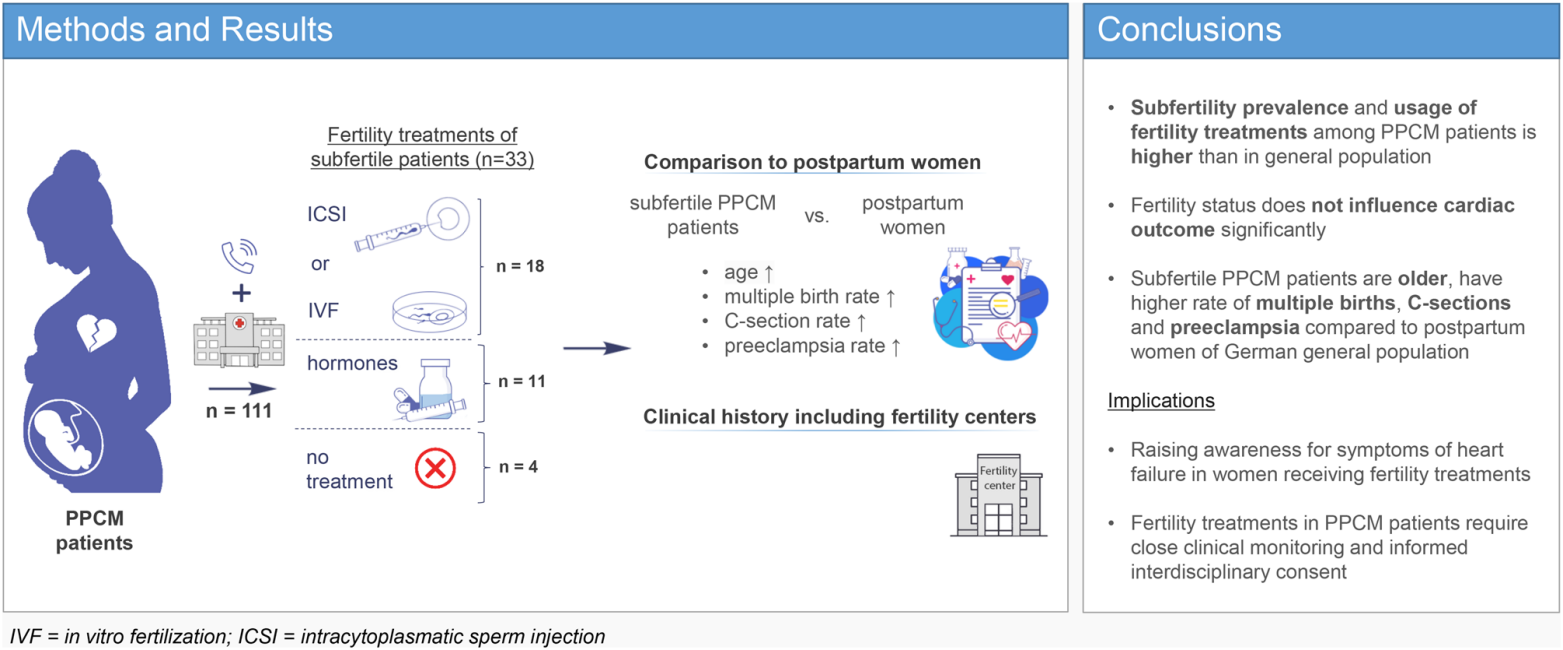

**Supplementary Information:**

The online version contains supplementary material available at 10.1007/s00392-022-02034-x.

## Introduction

In industrialized countries the pregnancy rate among young women is falling due to a wider usage of long-acting reversible contraceptives (LARCs), emergency contraceptives and changes in the social structure, leading to a rising maternal age at first birth [1]. Fertility of women decreases with increasing age requiring more frequently assisted reproductive technologies (ART) such as in vitro fertilizations (IVF) and, since male fertility decreases as well, intracytoplasmic sperm injections (ICSI) [2, 3]. Simultaneously, insurance coverage of ARTs expanded in various countries, contributing to an increasing use of ARTs in the past decades [4–6]. In Germany, the rate of born infants conceived by IVF/ICSI is 2.2% whereas in other European countries this rate ranges from 0.9% in Malta to 6.4% in Denmark (in 2014) [4]. In the United States the rate of infants conceived by IVF/ICSI has been calculated to be 1.6% in 2014 [7] and 1.8% in 2016 [5].

Pregnancies conceived or supported by ART procedures are associated with higher rates of hypertensive disorders such as preeclampsia [8]. Hypertensive disorders in turn are well-known risk factors for the development of peripartum cardiomyopathy (PPCM) [9]. PPCM is a life-threatening heart disease with onset in the last months of pregnancy, during delivery or in the first months after delivery in previously heart-healthy women [10]. PPCM is defined by heart failure due to left ventricular (LV) systolic dysfunction (LV-ejection fraction < 45%) [11, 12].

Hereby, we examine the prevalence of subfertility and ART procedures in a cohort of patients from the German PPCM registry in comparison to postpartum women in the German general population. In addition, we compared, outcome and potential risk factors including genetic risk factors and comorbidities in PPCM patients with and without subfertility and ART procedures.

## Methods

### Data collection

This study was approved by the local ethics committee of Hannover Medical School, Hanover, Germany (7970_BO_K_2018). The study complies with the Declaration of Helsinki and all patients and postpartum healthy controls gave written informed consent.

All PPCM patients who were treated at Hannover Medical School between January 2005 and March 2019 were screened for subfertility. Data regarding fertility status and applied subfertility treatment were collected via phone survey or in our PPCM outpatient clinic (Suppl. Figure 1). In this study, diagnosis of subfertility was based on either reported use of ART procedures or on reported failure to achieve a pregnancy after more than six months of regular unprotected sexual intercourse. The results were discussed and reviewed by gynecologists for reproductive medicine, who were blinded for the analysis of the medical findings.

Medical data at diagnosis, such as clinical signs of heart failure, echocardiographic parameters, laboratory assessments, drug treatment, adverse events and medical history, were collected. Follow-up (FU) data were obtained after 6 months (range 16–32 weeks). Very few data sets are incomplete in this registry as index PPCM was partly diagnosed in remote community hospitals or in an outpatient clinic from which patients were referred.

For a better evaluation of the outcome, we divided our collective in three subgroups, mainly depending on the LVEF at six months FU, as it has already been established in former studies [13]. In brief, full recovery was defined by an LVEF of ≥ 50%, partial recovery by an LVEF of 35–49% and no recovery was defined by an LVEF < 35% or the occurrence of an adverse event (implantation of a left ventricular assist device (LVAD), heart transplantation (HTX) or death).

To compare the prevalence of IVF/ICSI in our collective with the prevalence in the general population, the percentage of born infants conceived by IVF/ICSI among the total number of born infants of all interviewed patients was calculated for both collectives, using data from the Statistical Office of Germany and the German IVF-registry [14, 15]. Numbers were pooled for 2002 up to 2017 as the first live birth in our PPCM collective was in 2002 and the latest available data for the general population reaches up to 2017. These analyses were performed in cooperation with our local institute for biometrics.

### Exome sequencing and variant classification

Exome sequencing was performed on all PPCM patients with conducted IVF or ICSI whose blood samples were available. Furthermore, all PPCM patients with reported subfertility and history of cancer were analyzed. DNA was extracted from whole blood samples. DNA enrichment and library preparation were performed using the xGen® Exome Research Panel (Integrated DNA Technologies, Inc., Coralville, USA). Sequencing was performed on an Illumina NextSeq 500 using the NextSeq 500/550 High Output v2 kit (Illumina, San Diego, CA). Alignment to the reference genome build (GRCh37) was performed using megSAP, version 0.1-710-g52d2b0c (https://github.com/imgag/megSAP). Variant prioritization and visualization was performed with GSvar, version 2018_04 (https://github.com/imgag/ngs-bits), IGV [16], version 2.4.14 and with Alamut® visual, version 2.11 (Interactive Biosoftware, Rouen, France). Variants were classified according to the criteria proposed by the American College of Medical Genetics and Genomics (ACMG) [17], and 248 genes were analyzed per patient. Genes associated with dilated cardiomyopathy (DCM) were selected using the human phenotype ontology database [18] term “dilated cardiomyopathy” (HP:0001644; 115 genes); genes associated with DNA damage response (DDR) and general cancer predisposition syndromes (CPS) were composed of all genes listed in a benchmark study regarding cancer predisposition gene testing in adult patients [19] (Suppl. Table 1 and 2 for detailed information). Only genetic variants which were classified as ACMG class 4/5 (likely pathogenic/pathogenic) were considered. Part of the genetic data published in this study where already published and discussed in a former study, investigating the connection between PPCM and cancer [20].

### Statistical analysis

Data were analyzed using GraphPad Prism version 5.0f for Mac (GraphPad Software, La Jolla California, USA). Continuous data are expressed as mean ± SD or median (IQR) and categorical data as frequencies (%). Normal distribution was assessed using D'Agostino & Pearson omnibus normality test. Fisher’s exact test or Chi-square test was used for discrete variables depending on sample size, unpaired t test or Mann–Whitney U test were used for continuous variables. For multiple group comparison we used Chi-square test for discrete variables and one-way non-parametric ANOVA (Kruskal–Wallis test) for non-normally distributed continuous variables. A p-value of less than 0.05 was considered statistically significant.

## Results

### Prevalence of ART and infants conceived by IVF/ICSI in PPCM patients compared to the general population in Germany

**Fig. 1:**
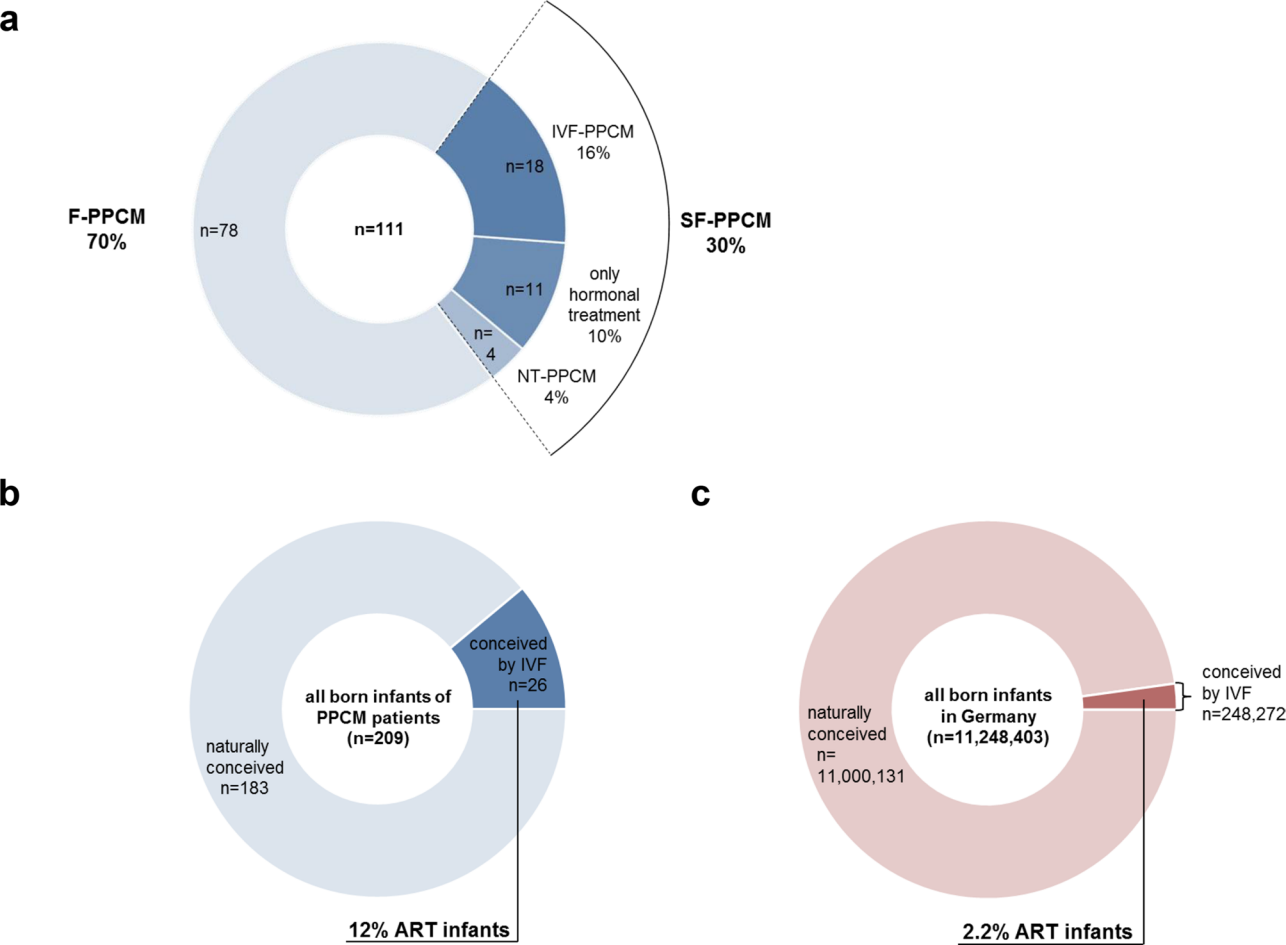
Distribution of PPCM patients in the different subgroups depending on fertility status and applied treatment protocol (**a**) and proportion of infants conceived by IVF/ICSI (ART infants) among PPCM patients (**b**) and in the German general population for the time period 2002–2017 (**c**)

In the present study, *n* = 111 patients with confirmed diagnosis of PPCM and known fertility status were included. Out of these, 30% (33/111) reported subfertility and/or received fertility treatment (SF-PPCM). Any kind of fertility treatment (ST-PPCM) was received by 26% (29/111) of all PPCM patients and 16% (18/111) were treated with IVF or ICSI (IVF-PPCM) (Fig. 1a). Among the 18 patients of IVF-PPCM, 16 patients were treated with IVF or ICSI prior to the index pregnancy, leading to the diagnosis of PPCM, and two patients underwent IVF/ICSI 45 or 17 months before delivery of index pregnancy. There were four patients with subfertility who did not receive any fertility-related treatment (NT-PPCM). In the present PPCM cohort 12% (26/209) of all born infants were conceived by IVF/ICSI. Currently, the percentage of infants conceived by IVF/ICSI in the general population in Germany is about 2.2% [14, 15] within the same time period, which is significantly (*p* < 0.0001) lower than in the PPCM cohort (Fig. 1b, c).

### Age, gravidity, parity and cardiac function in PPCM patients with and without subfertility

The *n* = 111 PPCM patients presented with a mean LVEF of 28 ± 10% at diagnosis. The median parity in all 111 patients was 1 (range: 1–6, Table 1). In one patient, index pregnancy resulted in a stillbirth, whereas 110 patients had pregnancies with live births. Parity was significantly lower in SF-PPCM compared to PPCM patients with unimpaired fertility (F-PPCM) (1.3 ± 0.7 vs. 1.8 ± 1.1; *p* = 0.0240) and the twin pregnancy rate was significantly higher (36% vs. 15%; *p* = 0.0221). All other baseline parameters were comparable between both groups including all parameters associated with severity of the disease such as NYHA class, LVEF, and NT-proBNP levels (Suppl. Figure 2). Furthermore, we compared the baseline parameters of our cohort with the baseline parameters of peripartum women in the normal population in Germany. PPCM patients were significantly older (34 ± 4 years vs. 31 ± 5 years; *p* < 0.0001), and had a significantly higher rate of multiple births (22% vs. 1.7%; *p* < 0.0001), C-sections (68% vs. 29%; *p* < 0.0001) and preeclampsia (31% vs. 2.6%; *p* < 0.0001). In contrast, smoking status (33% vs. 37%; ns) and parity (1.6 vs. 1.4; ns) were comparable between both groups and the prevalence of gestational diabetes was significantly lower in PPCM patients (6% vs. 13%; *p* < 0.0.05).

**Table 1:** Baseline characteristics of PPCM patients with and without subfertility

Baseline characteristics	All PPCM (*n* = 111)	Subfertile PPCM (SF-PPCM; *n* = 33)	Subgroup with IVF/ICSI (IVF-PPCM; *n* = 18)	Fertile PPCM (F-PPCM; *n* = 78)
Age in years	34 ± 4	35 ± 5	36 ± 4*	34 ± 4
Gravidity	1 (1–7)	1 (1–4)	1 [1–4]*	2 (1–7)
Parity	1 (1–6)	1 (1–4)*	1 (1–4)*	1 (1–6)
Twin pregnancy	22% (24/111)	36% (12/33)*	50% (9/18)*	15% (12/78)
C-Section	68% (71/104)	82% (27/33)	94% (17/18)*	62% (44/71)
Time from birth to first echo in days	33 ± 54	23 ± 48	17 ± 30	38 ± 57
Medical history				
Hypertension	13% (14/109)	9% (3/33)	0% (0/18)	14% (11/76)
Diabetes	4% (4/109)	3% (1/33)	6% (1/18)	4% (3/76)
(Former) nicotine abuse	33% (31/94)	27% (8/30)	13% (2/15)	36% (23/64)
History of cancer in total	11% (12/111)	24% (8/33)	17% (3/18)	5% (4/78)
Cancer before PPCM	7% (8/111)	18% (6/33)	17% (3/18)	3% (2/78)
Cancer after PPCM	5% (5/111)	9% (3/33)	0% (0/18)	3% (2/78)
Pregnancy-related conditions				
Preeclampsia	31% (34/111)	30% (10/33)	44% (8/18)	31% (24/78)
Gestational diabetes	6% (7/110)	6% (2/33)	6% (1/18)	6% (5/77)
Heart failure medication				
D2-agonist	87% (92/106)	94% (31/33)	94% (17/18)	84% (61/73)
ACE inhibitor	77% (82/106)	79% (26/33)	78% (14/18)	77% (56/73)
AT1-antagonist	18% (19/105)	15% (5/33)	17% (3/18)	19% (14/72)
MR-antagonist	74% (78/105)	61% (20/33)*	56% (10/18)	81% (58/72)
Beta-blocker	92% (99/108)	88% (29/33)	89% (16/18)	93% (70/75)
Diuretic	81% (86/106)	76% (25/33)	72% (13/18)	84% (61/73)

Continuous data are expressed as mean ± SD or median (range) according to normality of distribution. Dichotomous data are represented as percentage (number). Comparison between two groups (fertile group values were tested for statistical difference against subfertile group values or IVF/ICSI subgroup values) using unpaired *t*-test for metrical values with normal distribution, Mann–Whitney *U* test for metrical values with non-normal distribution and Fisher’s exact test for dichotomous values

IVF/ICSI, in vitro fertilization/intracytoplasmic sperm injection; LVEF, left ventricular ejection fraction; NT-proBNP, N-terminal pro-B-natriuretic peptide; NYHA, New York Heart Association; BL, baseline; D2-agonist, D2 dopamine receptor agonist; ACE inhibitor, angiotensin converting enzyme inhibitor; AT1-antagonist, AT1 angiotensin receptor antagonist; MR-antagonist, mineralocorticoid receptor antagonist

*p < 0.05. Numbers may not add up to 100% because of rounding

**Table 2:** Baseline characteristics of PPCM patients in comparison to peripartum women in the general population

Baseline characteristics	All PPCM (*n* = 111)	General population
Age in years	34 ± 4	31 ± 5**** [15]
Parity	1.63	1.41 [34]
Multiple births	22% (24/111)	1.7% (232,068/13,299,837)**** [35]
C-sections	68% (71/104)	29% (3,780,978/13,124,061)**** [36]
(Former) nicotine abuse	33% (31/94)	37% [37]
Preeclampsia	31% (34/111)	2.6% (3654/138,571)**** [38]
Gestational diabetes	6% (7/110)	13% (75,034/567,191)* [39]

Continuous data are expressed as mean ± SD or median (range) according to normality of distribution. Dichotomous data are represented as percentage (number)

As source data for parity and smoking status was not available, we could not perform statistical analysis comparing both groups for these properties. Both groups were compared using unpaired *t*-test for metrical values with normal distribution and Fisher’s exact test or Chi-square test for dichotomous values. *p < 0.05; ****p << 0.0001

Age in years for peripartum women in the German general population was calculated as pooled mean of ages for every woman’s birth in Germany from 2009 to 2018 whereas for PPCM patients only years of births of the PPCM pregnancies were included. All other data from the Statistical Office of Germany (parity, multiple births, C-sections) were pooled for the time period 2000–2018 analogous to the range of date of diagnosis in our questioned collective. The rate of (former) nicotine abuse for German general population factors in data from the German micro-census 2017 including women of age 20–45 (corresponding to the age range of 23–43 in our questioned collective)

### Causes of subfertility

In 45% (15/33) of SF-PPCM predominantly female causes of subfertility could be identified. Of those, ovulatory dysregulations presenting with menstrual dysfunction were reported in 44% (14/32), polycystic ovary syndrome (PCOS) in 12% (4/33), history of hyperprolactinemia in 19% (6/32), hypothyroidism in 45% (15/33) and diminished ovarian reserve defined by an anti-Müllerian hormone level below respective reference value in 18% (2/11). Organic causes such as endometriosis (12%, 4/33), tubal/ovarian abnormalities (22%, 7/32), uterus abnormalities (6%, 2/32) or past surgical procedures on the uterus/fallopian tubes/ovaries (34%, 11/32) were also present in SF-PPCM. Further risk factors that were present in our collective and that have shown to have an impact on subfertility, were advanced age (≥ 40 years) at start of successful fertility treatment (15%, 5/33), overweight defined by body mass index ≥ 25 kg/m^2^ (31%, 10/32), (former) nicotine abuse (27% 8/30), history of radio-/chemotherapy (15% 5/33) and heterozygous Fragile X syndrome (3%, 1/33) [21, 22].

In 18% (6/33) a predominantly male causation, indicated by changes in the concerned men’s spermiograms, and in another 18% (6/33) a combined contribution of both partners to the subfertility was assumed (Suppl. Table 3).

In 15% (5/33) no apparent cause neither in the female patients nor in their partners were found. One patient (3%, 1/33) conducted a prophylactic cryopreservation prior to chemotherapy and was included in our analysis analogous to the definition of our inclusion criteria given above (Table 2).

### Fertility treatment protocols applied to PPCM patients

For further analyses, only fertility treatment regimens applied less than three months before conception of index PPCM were included (*n* = 24). Sixteen patients underwent an ART procedure directly prior to the index pregnancy. Out of these, nine patients obtained ICSI and six patients underwent IVF without intracytoplasmic injection. For one patient no data from the treating fertility center with regard to the fertility procedure was available.

Nine patients underwent controlled ovarian hyperstimulation (COH) directly prior to the embryo transfer with seven patients obtaining a long protocol with administration of gonadotropin-releasing hormone (GnRH)-agonists and two patients obtaining a short protocol with administration of GnRH-antagonists. Five patients underwent an embryo transfer in a cryo-thaw cycle without any additional medication for that cycle and the remaining two patients got pregnant after ovum donation.

For luteal support, progesterone was administered in 91% (20/22) and estrogen was given in 45% (10/22) of the ST-PPCM patients. Human chorionic gonadotropin (hCG) was utilized for ovulation induction in 74% (17/23) of these PPCM patients. For ovarian stimulation, follicle-stimulating hormone (FSH) was administered in 52% (12/23), human chorionic gonadotrophin (hMG) in 13% (3/23) and clomiphene in 9% (2/23) for the same objective. These numbers vary if applied medications of previous unsuccessful treatment cycles are included (Suppl. Figure 3).

### Genetic analysis

Since genetics contribute to both, PPCM and subfertility, exome sequencing was performed in a subset of *n* = 15 SF-PPCM patients with reported subfertility. Out of these, 12 patients received fertility treatment. In five out of these 15 patients (33%), either pathogenic (P, class 5) or likely pathogenic (LP, class 4) variants associated with dilatative (DCM) or hypertrophic (HCM) cardiomyopathies (*n* = 5) and/or cancer predisposition syndrome (CPS, *n* = 3), with two patients carrying mutations associated with both pathologies, were found (Suppl. Table 1 and 2). Of note, eight patients of SF-PPCM had a history of cancer prior to (*n* = 6) or after (*n* = 3) PPCM (one patient suffered from both, cancer prior to and after PPCM) and 88% (7/8) of the pathogenic or likely pathogenic mutations were found in patients with a history of cancer.

### Impact of subfertility and fertility treatments on the cardiac outcome in PPCM patients

**Fig. 2:**
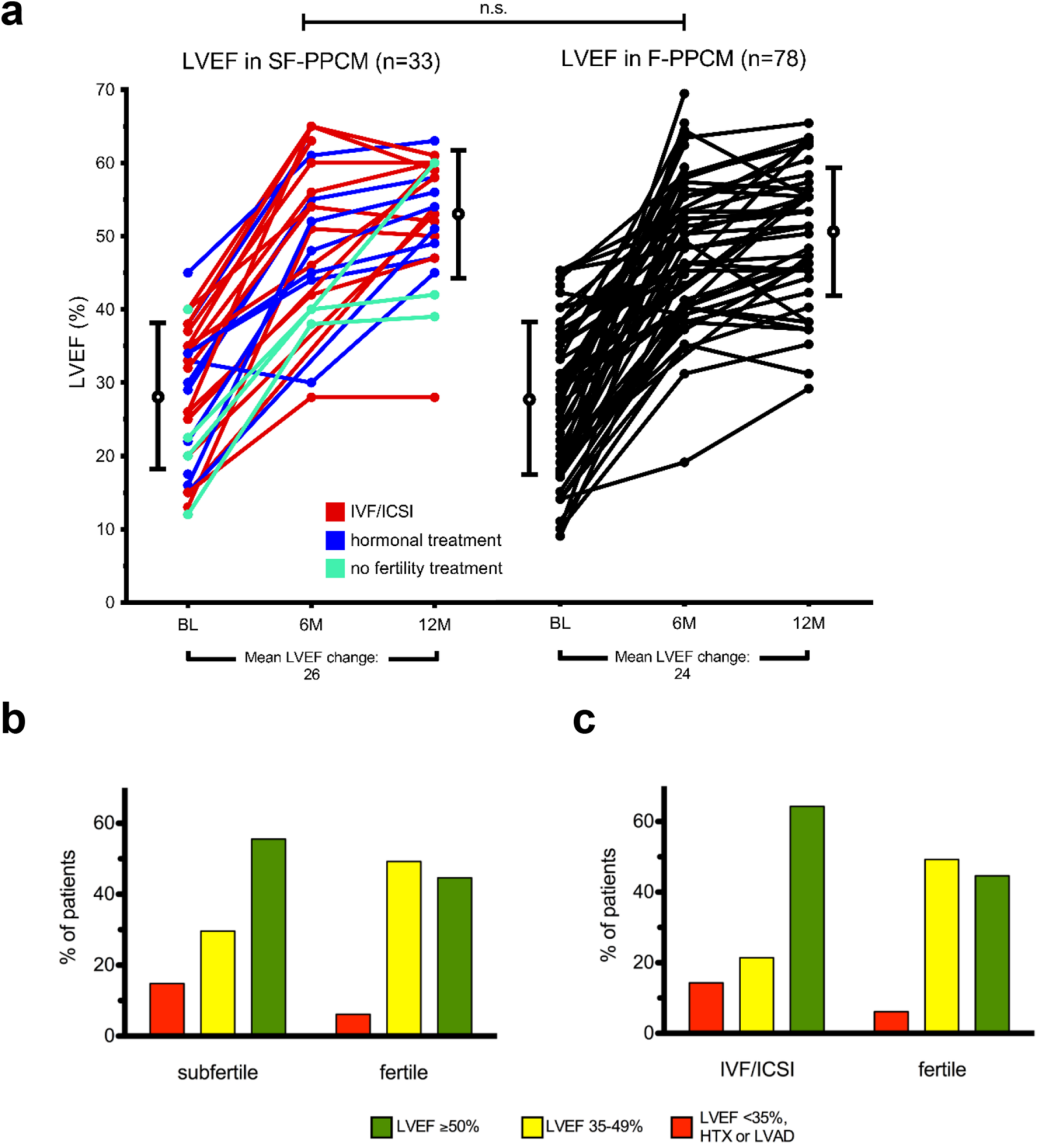
Course of LVEF in SF-PPCM and F-PPCM at baseline, 6 and 12 months follow-up (**a**) and recovery status at 6 months follow-up of SF-PPCM and F-PPCM (**b**) and of IVF-PPCM and F-PPCM (**c**). BL, baseline; LVAD, left ventricular assist device; LVEF, left ventricular ejection fraction; HTX, heart transplantation; IVF/ICSI, in vitro fertilization/intracytoplasmic sperm injection; M, months. Red column: non-recovery (LVEF ≤ 35%, HTX, LVAD-implantation or death); yellow column: partial recovery (LVEF > 35-49%); green column: full recovery (LVEF ≥ 50%). Chi-square tests were performed on the distribution of the three recovery groups and did not retrieve any statistically significant results

To evaluate the impact of subfertility on the clinical course of PPCM, we measured LVEF and NT-proBNP-levels 3, 6 and 12 months after diagnosis. At all time points, LVEF as well as NT-proBNP levels did not differ significantly between F-PPCM and SF-PPCM (Fig. 2a and Suppl. Figure 2a, b). Furthermore, mortality (0% vs. 0%) and the rate of implanted LVAD (3% vs. 1%, 1/33 vs. 1/78) or HTX (3% vs. 0%, 1/33 vs. 0/78) were similar in both groups.

Moreover, the recovery status at 6 and 12 months FU did neither differ between SF-PPCM and F-PPCM nor between IVF-PPCM and F-PPCM (Fig. 2b, c).

## Discussion

The present study is the first to address subfertility and the high rate of conducted ART procedures among PPCM patients in depth. A risk factor analysis of an Israeli cohort of 36 PPCM patients revealed a high rate of patients conceiving by IVF [23], which we also observed in our larger cohort of 111 German PPCM patients. The use of ARTs, especially IVF/ICSI procedures, was significantly higher in PPCM patients compared to the general population in Germany.

In our study cohort, well-described risk factors for both PPCM and subfertility such as high maternal age and hormonal disorders, were elevated compared to pregnant women in the general population. These risk factors were also more prevalent than in PPCM collectives with different ethnical backgrounds. In the largest African PPCM registry, the PEACE Registry [24], PPCM patients were younger, parity was higher and hormonal disorders were less frequent compared to our German PPCM collective. These distinctions go in line with the findings from the EORP registry, the largest international registry including 739 women with PPCM from 49 different countries [10]. Therein, European PPCM patients were older and had a lower number of previous pregnancies compared to PPCM patients from Africa, Asia–Pacific or Middle East, suggesting a distinct set of risk factors for individual ethnic groups. Interestingly, hormonal disorders were more frequent in the PPCM cohorts from Asia and Middle East. In comparison to the largest Asian PPCM study from Taiwan with 925 PPCM patients [25], our German PPCM collective displayed a higher maternal age, a higher multiparity rate and a similar rate of previous deliveries (33% vs. 36%). To evaluate the role of ethnic backgrounds as well as the accessibility and usage of assisted reproductive technology in other countries with regard to an increased risk for PPCM, our findings should be validated in a larger, ethnically diverse cohort of PPCM patients.

In addition, twin pregnancies, which occur more commonly after IVF/ICSI due to general practice of multiple embryo transfers, are a known risk factor for PPCM [26]. Indeed, in the present cohort, twin pregnancy rate was higher in PPCM patients with ART compared to PPCM patients with naturally conceived pregnancies. With regard to the causes for the observed higher incidence of subfertility in PPCM patients, subfertile PPCM patients frequently carried pathogenic or likely pathogenic gene variants associated with either DCM/HCM and/or CPS, which may impact fertility due to lower rate of functional eggs and increased rate of early abortion due to genetic impairment in embryos. In addition, some of the subfertile PPCM patients underwent anticancer treatment prior to the index pregnancy, which can also impair fertility [27] and can lead to a higher risk for cardiomyopathies [20] at the same time. Thus, the anticancer treatment may have also contributed to the connection between subfertility and PPCM.

Furthermore, shared pathophysiological pathways could also play a role for the observed high prevalence of subfertility and ART in PPCM patients. For example, excessive oxidative stress is known to play a key role in the pathophysiology of PPCM [28, 29] and has also been described as a key factor in subfertility-associated diseases such as endometriosis [30] and polycystic ovary syndrome (PCOS) [31, 32].

Interestingly, both subfertility and the use of ART procedures did not affect the clinical course of PPCM, as the cardiac outcome was comparable between subfertile and fertile PPCM patients, suggesting that neither the condition nor the ART treatment are influencing the course of the disease.

Concluding from these data, women with subfertility problems, especially when receiving fertility treatment, should be closely monitored for cardiovascular complications in the peripartum phase. Suspicious signs of heart failure like dyspnea should be taken seriously and lead to early cardiac examination to rule out PPCM. Peripartum determination of NT-proBNP could serve as a biomarker for cardiac health in women who conceived by IVF/ICSI and thus help to diagnose PPCM early.

Patients with diagnosed PPCM and a history of subfertility who are planning a subsequent pregnancy and seek medical assistance (especially ART) should consult their treating physician and should be carefully informed about the potential risks. In these patients, ART procedures should only be conducted as result of a risk–benefit analysis after informed consent between cardiologist, gynecologist and the patient. Once pregnant, these patients should remain under close clinical monitoring whereby the use of biomarkers such as NT-proBNP and echocardiographic examinations may help to identify worsening of cardiac function during and after pregnancy. In this context it is important to note that PPCM can also occur after early abortions.

Further studies are needed to examine the connection of PPCM and subfertility more closely and to elucidate the pathophysiological mechanism behind the accumulation of subfertility among PPCM patients.

## Limitations

Not all patients included in this study were diagnosed in our hospital and thus baseline data are partly incomplete. For the same reason we were not able to retrieve blood samples at baseline from all PPCM patients.

The definition of subfertility varies in the literature as much as the concept itself is not widely recognized as a narrowly defined term. The time to pregnancy is a subjective measure to estimate the severity of subfertility. We were guided by a German study for subfertility that determined the lightest form of impaired fertility at 6 months of unsuccessful conception [33].

## Conclusions

Subfertility and fertility treatments are more frequent in PPCM patients compared to the general population. It remains unclear whether fertility treatments are an underlying risk factor for the development of PPCM or whether these patients carry risk factors and pathophysiological conditions that cause both, subfertility and a predisposition for PPCM. Whole exome sequencing revealed an increased number of mutations in PPCM patients with subfertility, but these mutations occurred mainly in PPCM patients with history of cancer, making genetic alterations an improbable connection between these two entities. The cardiac outcome did not differ significantly between PPCM patients with and without subfertility.

Women receiving fertility treatment should be closely monitored for symptoms of heart failure and should be immediately referred to a cardiologist in case of suspected PPCM. PPCM patients who are planning a subsequent pregnancy should only consider fertility treatment under close clinical monitoring and after informed consent between cardiologist, gynecologist and patient.

## Supplementary Information

Below is the link to the electronic supplementary material.

Supplemental Fig. 1 Study flow diagram of our analysis of subfertility and fertility treatments among PPCM patients. ART, assisted reproductive technology. Supplemental Fig. 2 LVEF (a) and NT-proBNP (b) in the respective groups of questioned PPCM patients at baseline, 3, 6 and 12 months follow-up. NYHA class at 6 months follow-up for the same groups (c): BL, baseline; FU, follow-up; IVF/ICSI, in vitro fertilization/intracytoplasmic sperm injection; LVEF, left ventricular ejection fraction; M, months; NT-proBNP, N-terminal pro-B-natriuretic peptide; NYHA, New York Heart Association. LVEF and NT-proBNP values at the different time points of each group were compared using one-way non-parametric ANOVA (Kruskal-Wallis test) and for the comparison of the distribution of FU NYHA class in the different groups a Chi-square test was carried out. Neither of the tests revealed any statistically significant results. Supplemental Fig. 3 Applied fertility treatment medication within 3 months prior to index pregnancy and in total. GnRH (gonadotropin-releasing hormone); FSH (follicle-stimulating hormone); LVEF (left ventricular ejection fraction); LH (luteinizing hormone); hMG (human menopausal gonadotropin); hCG (human chorionic gonadotropin) (PPTX 671 kb)

Supplementary file2 (PDF 281 kb)
