# Supplementary figures and images for: High prevalence of reduced fertility and use of assisted reproductive technology in a German cohort of patients with peripartum cardiomyopathy
Source: Clin Res Cardiol. 2022 May 13;112(3):343–52. doi: 10.1007/s00392-022-02034-x (PMC9998571; doi:10.1007/s00392-022-02034-x)

## Slide 1
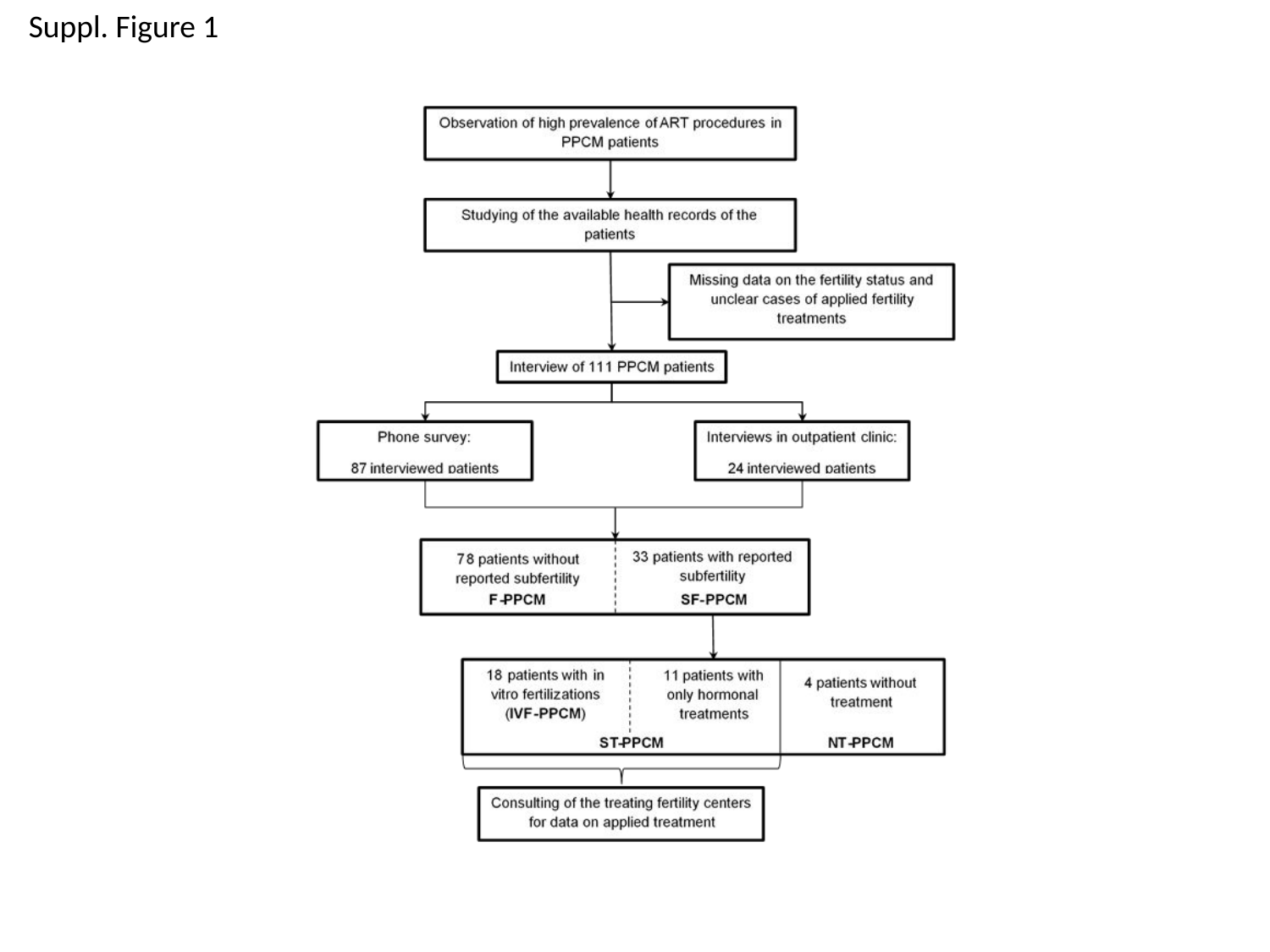

Suppl. Figure 1

## Slide 2
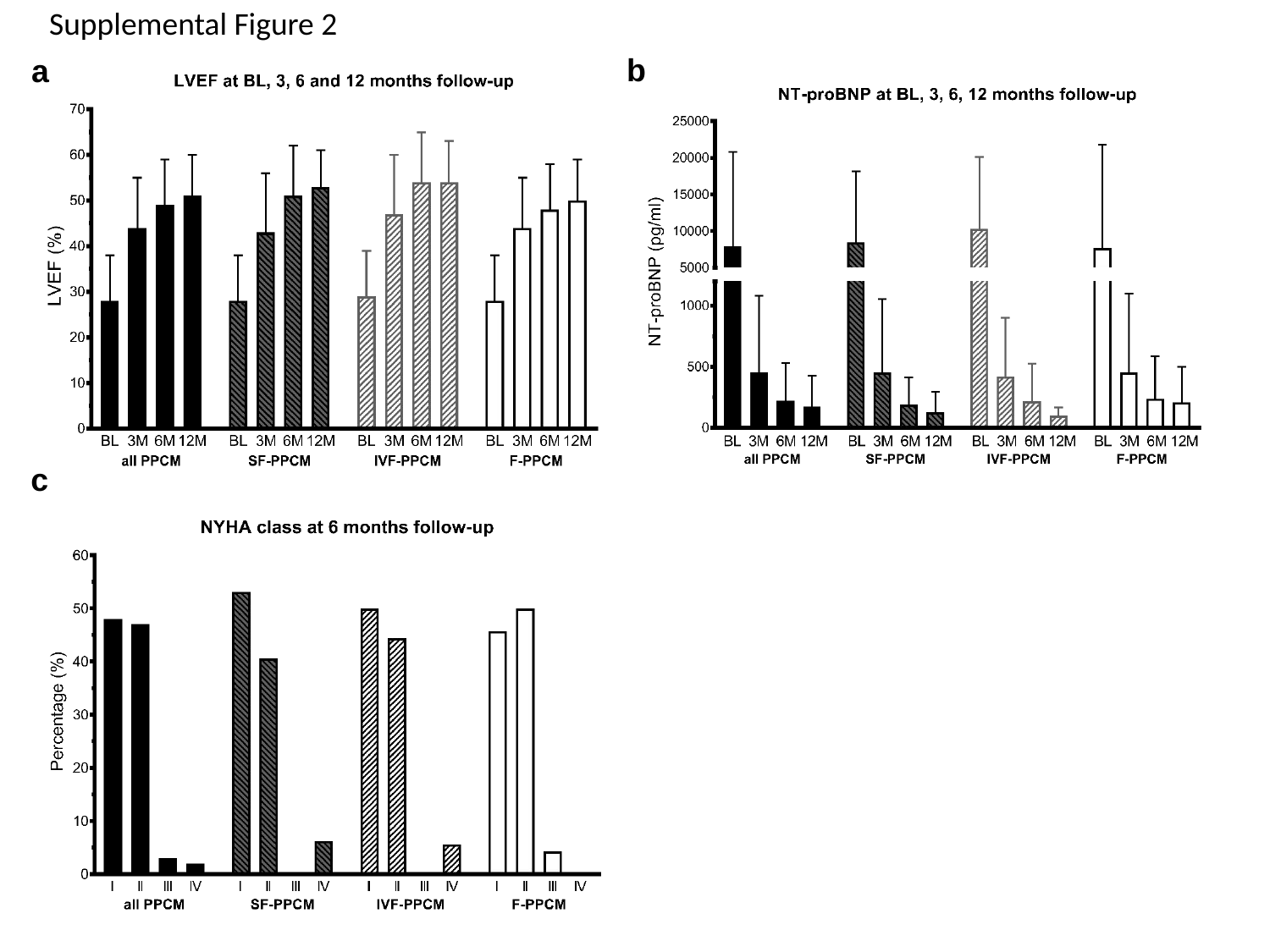

Supplemental Figure 2
b
a
c

## Slide 3
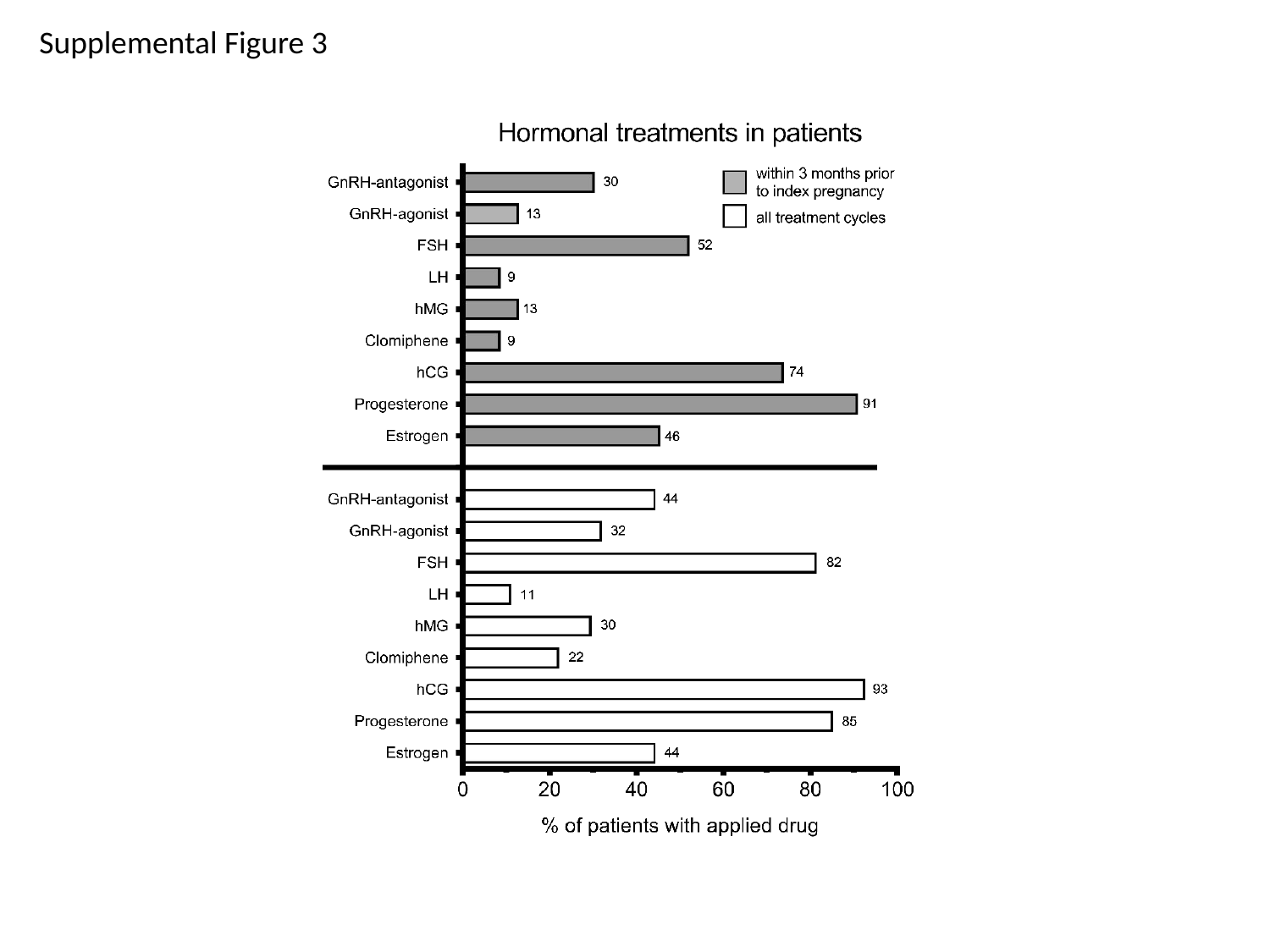

Supplemental Figure 3

Supplement: Supplementary file 1 — Supplemental Fig. 1 Study flow diagram of our analysis of subfertility and fertility treatments among PPCM patients. ART, assisted reproductive technology. Supplemental Fig. 2 LVEF (a) and NT-proBNP (b) in the respective groups of questioned PPCM patients at baseline, 3, 6 and 12 months follow-up. NYHA class at 6 months follow-up for the same groups (c): BL, baseline; FU, follow-up; IVF/ICSI, in vitro fertilization/intracytoplasmic sperm injection; LVEF, left ventricular ejection fraction; M, months; NT-proBNP, N-terminal pro-B-natriuretic peptide; NYHA, New York Heart Association. LVEF and NT-proBNP values at the different time points of each group were compared using one-way non-parametric ANOVA (Kruskal-Wallis test) and for the comparison of the distribution of FU NYHA class in the different groups a Chi-square test was carried out. Neither of the tests revealed any statistically significant results. Supplemental Fig. 3 Applied fertility treatment medication within 3 months prior to index pregnancy and in total. GnRH (gonadotropin-releasing hormone); FSH (follicle-stimulating hormone); LVEF (left ventricular ejection fraction); LH (luteinizing hormone); hMG (human menopausal gonadotropin); hCG (human chorionic gonadotropin) (PPTX 671 kb) [file 392_2022_2034_MOESM1_ESM.pptx]
