# Supplementary material for: High prevalence of reduced fertility and use of assisted reproductive technology in a German cohort of patients with peripartum cardiomyopathy
Source: Clin Res Cardiol. 2022 May 13;112(3):343–52. doi: 10.1007/s00392-022-02034-x (PMC9998571; doi:10.1007/s00392-022-02034-x)
Supplement: Supplementary file 2 — Supplementary file2 (PDF 281 kb) [file 392_2022_2034_MOESM2_ESM.pdf]

Supplemental Table 1: List of genes associated with the Human Phenotype Ontology term "dilated cardiomyopathy"

Pathogenic (P, class 5) or likely pathogenic (LP, class 4) variants associated with dilatative or hypertrophic cardiomyopathies that were found in the PPCM collective are highlighted in bold and italics (n=5 mutations as 3 different TTN mutations could be identified)

ABCC9	EYA4	MAP3K20	SDHAF1	VCL
CAD8	FHL1	MGME1	SDHD	XK
ACAD9	FHL2	MMP1	SELENON	XRCC4
CTA1	FKRP	MT-TK	SGCB	
ACTC1	FKTN	MYBPC3	SGCD	
ACTN2	GABRD	MYH6	SKI	
ADCY5	GATA5	MYH7	SLC25A4	
ALMS1	GATAD1	MYL2	SLC2A10	
ANKRD1	GLB1	MYPN	SPEG	
ANKRD11	HACD1	NDUFB11	SYNE1	
BAG3	HADH	NEBL	SYNE2	
BBS2	HADHA	NEXN	TAF1A	
BOLA3	HADHB	PGM1	TAZ	
CHKB	HAMP	PLN	TCAP	
COL7A1	HCCS	POLG	TMEM43	
COX7B	HJV	POLG2	TMPO	
CPT2	ITGA7	PPCS	TNNC1	

CRYAB	JUP	PRDM16	TNNI3
CSRP3	KAT6B	PSEN1	TNNI3K
DES	KCNAB2	PSEN2	TNNT2
DMD	LAMA3	RAF1	TPM1
DNAJC19	LAMA4	RBCK1	TPM2
DOLK	LAMB3	RBM20	TPM3
DPM3	LAMC2	RERE	TSFM
DSG2	LAMP2	RRM2B	***TTN***
DSP	LDB3	RYR1	TWNK
EPG5	LIMS2	SCN5A	TXNRD2
ERBB3	LMNA	SDHA	UBR1

Supplemental Table 2: List of genes associated with CPS (Whitworth et al., AJHG 2018, https://doi.org/10.1016/j.ajhg.2018.04.013)

Pathogenic (P, class 5) or likely pathogenic (LP, class 4) variants associated with cancer predisposition syndrome that were found in the PPCM collective are highlighted in bold and italics

ATM	ELANE	HMBS	PRSS1	SMARCB1
AXIN2	EPCAM	HNF1A	PTCH1	SMARCE1
BAP1	ERCC2	HRAS	PTEN	SOS1
BLM	ERCC3	ITK	PTPN11	SRY
BMPR1A	ERCC4	KIT	RAD51C	STAT3
BRCA1	***ERCC5***	MAX	RAD51D	STK11
BRCA2	EXT1	MEN1	RB1	SUFU
BRIP1	EXT2	MET	RECQL4	TERT
BUB1B	EZH2	MLH1	RET	TGFBR1
CBL	FAH	MSH2	RHBDF2	TMEM127
CDC73	FANCA	MSH6	RMRP	TP53
CDH1	FANCB	MTAP	RUNX1	TRIM37
CDK4	FANCC	MUTYH	SBDS	TSC1
CDKN1B	FANCD2	***NBN***	SDHA	TSC2
CDKN1C	FANCE	NF1	SDHAF2	UROD
CDKN2A	FANCF	NF2	SDHB	VHL

CDKN2B	FANCG	NSD1	SDHC	WAS
CEBPA	FANCI	NTHL1	SDHD	WRN
CEP57	FANCL	PALB2	SERPINA1	WT1
CHEK2	FANCM	PDGFRA	SH2D1A	XPA
COL7A1	FAS	PHOX2B	SLC25A13	XPC
CYLD	FH	PMS1	SLX4	
DDB2	FLCN	PMS2	SMAD4	
DICER1	GATA2	POLD1	SMARCA4	

Supplemental Table 3: Subfertility factors among SF-PPCM according to the fertility centers

**Possible causes of subfertility**	**Affected patients**
**Ovulatory dysregulation**	
Menstrual dysfunction	44% (14/32)
PCOS	12% (4/33)
History of hyperprolactinemia	19% (6/32)
Hypothyroidism	45% (15/33)
Diminished ovarian reserve (AMH below reference value)	18% (2/11)
**Structural causes**	
Endometriosis	12% (4/33)
Tubal/ovarian abnormalities	22% (7/32)
Uterus abnormalities	6% (2/32)
History of gynecologic operations on uterus/fallopian tubes/ovaries	34% (11/32)
**Other risk factors**	
Age ≥40 years at start of successful fertility treatment	15% (5/33)
High BMI (≥25kg/m²)	31% (10/32)

Nicotine abuse	27% (8/30)
History of radiotherapy or chemotherapy	15% (5/33)
Prophylactic cryopreservation before chemotherapy	3% (1/33)
Heterozygous Fragile X syndrome	3% (1/33)
**Male factor**	
Abnormal spermiogram of partner	67% (12/18)

AMH, anti-Müllerian hormone; BMI, body mass index; PCOS, polycystic ovary syndrome.
Dichotomous data are represented as percentage (number).
